# Suicides in state prisons in the United States: Highlighting gaps in data

**DOI:** 10.1371/journal.pone.0285729

**Published:** 2023-05-31

**Authors:** Katherine LeMasters, Michael F. Behne, Jennifer Lao, Meghan Peterson, Lauren Brinkley-Rubinstein

**Affiliations:** 1 Department of Epidemiology, Gillings School of Global Public Health, University of North Carolina at Chapel Hill, Chapel Hill, North Carolina, United States of America; 2 Center for Health Equity Research, Department of Social Medicine, School of Medicine, University of North Carolina at Chapel Hill, Chapel Hill, North Carolina, United States of America; 3 Department of Population Health Sciences, Duke University School of Medicine, Duke University, Durham, North Carolina, United States of America; University of Bristol, UNITED KINGDOM

## Abstract

**Objectives:**

Our objectives were to document data availability and reporting on suicide mortality in state prison systems. The United States leads the world in mass incarceration, a structural determinant of health, but lacks real-time reporting of prison health statistics. This absence is particularly notable in suicides, a leading cause of death that carceral policies play a key role in mitigating.

**Methods:**

Suicide data for each state prison system from 2017–2021 were gathered through statistical reports, press releases, and Freedom of Information Act requests. We graded states based on data availability.

**Results:**

Only sixteen states provide updated, frequent, granular, freely provided suicide data. An additional thirteen states provided frequently updated data but that had little granularity, was incomplete, or was not freely provided. Eight states provided sparse, infrequent, or outdated data, and thirteen provided no data at all.

**Conclusions:**

The 2000 Death in Custody Reporting Act requires that states provide these data freely, yet the majority of states do not. There is a need for reliable, real-time data on suicides, suicide attempts, and conditions of confinement to better understand the harms of the carceral system and to advocate for change.

## Introduction

Time in prison is associated with poor mental health both during and post-incarceration. High levels of stress, a loss of autonomy, removal from social networks and communities, inhumane and often unpredictable living conditions, and little to no mental health treatment access can create the perfect storm for mental health crises in prison [1, 2]. Historically, in the United States, the deinstitutionalization of those with mental illness often results in those without access to mental health treatment spending time behind prison walls [3]. Post-release, the lack of linkage to mental healthcare, worsening of mental health in prison, and the onslaught of competing needs and priorities (e.g., housing, health insurance, employment, food security) creates a vicious cycle—contributing to even worse mental health, high post-release mortality by suicide and other causes, and individuals cycling back into jail and prison [4–6]. Furthermore, these mental health consequences are inequitably distributed throughout the population. Incarceration is disproportionately concentrated among Black individuals with a high school education or les, living in historically disinvested communities due to the criminal legal system’s roots in structural racism [7, 8]. This should all serve as a red flag for public health experts, advocates, and policy makers to pay attention to mental health indicators—particularly suicide—among incarcerated individuals.

While suicides in-custody have increased over time and are a leading cause of death in prisons, reporting on the national level by the Bureau of Justice Statistics lags approximately three years behind [9]. While empirical studies and reporting have long-found that prisons create long-lasting effects on mental health, it is critical that we are able to document conditions of confinement and suicides at the national level to hold Departments of Corrections (DOCs) accountable and advocate for change. The lag in reporting has been particularly noticeable during the COVID-19 pandemic, a time in which mental health has likely worsened behind prison walls and people were exposed to extreme conditions to mitigate disease. For example, while solitary confinement (a practice in which incarcerated individuals are housed alone for 23 or more hours and is characterized by extreme conditions of isolation, sensory deprivation, and idleness) [10, 11] is a generally used as a disciplinary sanction, it was also used for social control and disease containment during the COVID-19 pandemic [12]. Furthermore, it is well-documented that mental health worsened in the general population due to increased stress, anxiety, and isolation [13]. Those in prison faced the same stressors while also experiencing frequent, large-scale COVID-19 outbreaks and an inability to practice social distancing or access personal protective equipment. Yet, mental health and suicides in prison during COVID-19 remains undocumented. Therefore, our objective was to document data availability on suicide mortality in prisons during this critical time.

## Methods

Data on suicides in-custody should be publicly available via the 2000 Death in Custody Reporting Act, but they often are not [14]. Third City Project was created to democratize data on carceral systems and health, with its first aim being to document mortality and cause of death data, including suicide deaths, from state prison systems [15]. Our team searched the 50 United States’ DOC websites for statistical reports (e.g., reports that are freely available on DOC websites) and public-facing press releases for this information. Thus, all data come from state prison systems (e.g., facilities run by state jurisdiction that typically houses people sentenced to over a year of incarceration) rather than federal prisons or jails (e.g., facilities run by a city, local district, or county that typically houses people sentenced to less than a year of incarceration). All information recorded on deaths was extracted into a REDCap form and double-coded with a third coder reconciling differences. We also submitted Freedom of Information Act (FOIA) requests for all state prison systems for deaths. We searched statistical reports and press release information and sent FOIA requests for data from years 2000–2021. Press release data collection took place from October 2021 through August 2022. Statistical report data searches took place from November 2021 until March 2022. FOIA requests were sent between May and June 2021. FOIA requests that required payment upon delivery of responsive documents and those that took longer than four months to complete were not included in the analysis.

We assessed data recency, data reporting frequency (e.g., monthly, yearly), data granularity (e.g., system-wide, facility-level, individual-level), context availability (e.g., housing status at time of death, suicide attempts), and data source (e.g., freely provided through statistical report or press release, FOIA). We graded each state prison system by level of data availability (F: no data, C: sparse or infrequent data, particularly in recent years, B: frequently updated data but data had little granularity or was not freely provided, A: frequently updated and freely provided, granular data). More specifically, states received a C if they had reported data at some point but not from 2017/2018-2020/2021. States received a B if they provided data during this time but it was incomplete, only available annually, released only at the system level, or was only accessible via FOIA. States received an A if data were provided consistently, were complete, were available monthly or daily, were available at the facility or individual level, and were made freely available through statistical reports or press releases. States received a deduction of one letter grade if they initially received an A but there was evidence from additional sources that reported suicides were an undercount.

## Results

**Fig 1:**
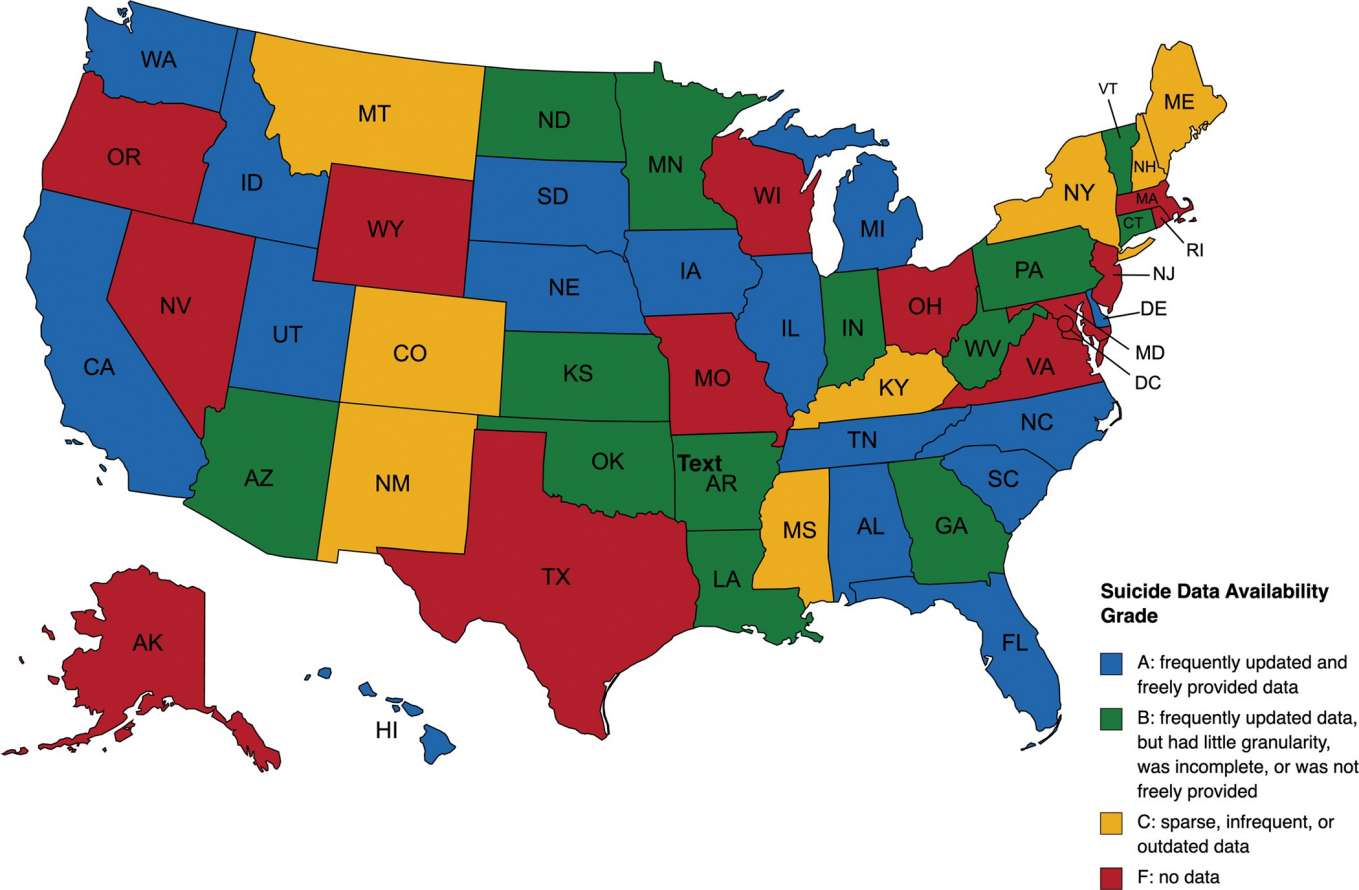
State map by prison system suicide data grade. Republished from MapChart under a CC BY license, with permission from MapChart, original copyright 2014.

Thirteen states provided no suicide data (received an F), eight states provided sparse, infrequent, or outdated data (received a C), thirteen states provided frequently updated data but that had little granularity, was incomplete, or was not freely provided (received a B), and sixteen states provided frequently updated, freely provided, granular data (received an A) (**Tables 1 and 2; Fig 1**). Only eight states provided data that included 2021, with the majority of those reporting only providing data through 2020. Of the 37 states providing data, 16 provide data annually, seven monthly, and 25 provide individual data, which includes date of death. Some states provide data in multiple forms with California and Illinois being the only states to provide data at all three frequencies. Eighteen states report system-level data, sixteen report facility-level data, and 25 individual-level data. The 25 states reporting individual data provide the most granular data, as these data often include the individuals’ age, race, ethnicity, and more specific information on the cause of death. Only three states (California, Florida, Kansas) provide data on housing status (e.g., solitary confinement, security level) at the time of death. Six states (Alabama, Arizona, Arkansas, Connecticut, Michigan, Tennessee) report suicide attempts. Twenty-three states provided data freely via statistical reports or press releases and 13 responded to FOIA requests.

**Table 1:** Suicide data grading by United States state prison system.

	Data Recency		Data Frequency		Data Level			Additional Information		Data Source			Data Grade***
State	Any Suicide Data	Data in Recent Years (2017/2018-2020/2021)	Yearly	Monthly	System	Facility	Individual+	Housing Status++	Suicide Attempts	FOIA	Statistical Reports	Press Releases	
AL	X	X		X	X	X			X		X		A
AK													F
AZ*	X	X	X		X				X		X		B
AR	X	X		X	X				X		X		B
CA	X	X	X	X	X	X	X	X			X	X	A
CO	X		X		X						X		C
CT*	X	X	X		X				X		X		B
DE	X	X				X	X					X	A
FL	X	X	X		X		X	X			X	X	A
GA**+++	X	X				X	X					X	B
HI	X	X	X		X		X			X		X	A
ID**	X	X				X	X					X	A
IL	X	X	X	X	X		X			X	X		A
IN	X	X					X			X			B
IA	X	X				X	X					X	A
KS	X	X		X	X			X		X			B
KY	X						X			X			C
LA*	X	X	X		X		X				X	X	B
ME	X						X			X			C
MD													F
MA													F
MI	X	X	X	X		X			X	X	X		A
MN	X	X	X		X						X		B
MS	X		X		X						X		C
MO													F
MT	X		X								X		C
NE**	X	X				X	X					X	A
NV													F
NH	X					X	X					X	C
NJ													F
NM	X						X			X			C
NY	X		X		X						X		C
NC	X	X				X	X					X	A
ND	X	X	X		X		X			X			B
OH													F
OK*+++	X	X				X	X					X	B
OR													F
PA	X	X					X			X			B
RI													F
SC	X	X				X	X			X		X	A
SD**	X	X				X	X					X	A
TN*	X	X		X	X	X			X		X		A
TX													F
UT	X	X	X		X	X	X			X		X	A
VT*	X	X				X	X					X	B
VA													F
WA	X	X	X		X		X			X	X		A
WV	X	X					X			X			B
WI													F
WY													F

* Not full data, not used in analyses

** location of death provided in press release data, which provides facility information

+Indicates Daily Data

++ Solitary confinement status or security level given

+++ Evidence of suicide undercounts from additional sources

*** A: frequently updated and freely provided data; B: frequently updated data but that had little granularity, was incomplete, or was not freely provided; C: sparse, infrequent, or outdated data; F: no data

Source/Notes: Authors’ analysis of state Department of Corrections statistical reports, press releases, and Freedom of Information Act responses on suicide death reporting in custody.

**Table 2:** Summary of suicide data quality in United States state prison systems.

	N	%
**Data Recency**		
Any	37	74
Recent Years (2017/2018-2020/2021)	29	58
**Data Frequency**		
Yearly	16	32
Monthly	7	14
Daily	25	50
**Data Level**		
System	18	36
**Facility**	16	32
Individual	25	50
**Additional Information**		
Housing Status	3	6
Suicide Attempts	6	12
**Data Source**		
FOIA	13	26
Statistical Reports	16	32
Press Releases	15	30
**Data Grade**		
A	16	32
B	13	26
C	8	16
F	13	26

Source/Notes: Authors’ analysis of state Department of Corrections statistical reports, press releases, and Freedom of Information Act responses on suicide death reporting in custody.

## Discussion

Suicide rates have historically been a leading cause of death in prisons, yet real-time information on suicides is often unavailable. During the COVID-19 pandemic, prison conditions generally worsened, which included barring visitors and frequently altering individuals’ living conditions, which likely subsequently worsened mental health [16]. However, in order to advocate for change, we must first understand the mental health burden behind prison walls.

Thirteen states provided no data on suicides and an addition seven have provided sparse or infrequent data insufficient for understanding suicide trends or alerting the public health community to mental health crisis. This is despite the 2000 Death in Custody Reporting Act requiring that states provide this data freely and our team submitting FOIA requests for such data. These gaps suggest a need for real-time data on both suicides and suicide attempts across systems. Suicide attempt data can serve as an important indicator for severe mental health needs, especially as systems do not report on other mental health concerns [17]. This is an area of critical need for public health experts and policymakers given the high amount of suicides in prisons.

Historically, official reports from DOC are also often a large undercount of suicides and recent reports from news sources indicate that suicides are rapidly increasing. For example, The Atlanta Journal-Constitution documented 125 suicides since the beginning of 2017 in Georgia state prisons from a combination of DOC documents, death certificates, additional records [18]. In addition, in multiple states, DOCs do not report suicides in a systematic way (e.g., FOIA, statistical reports, press releases), but news articles have documented suicides in these state DOCs [19–21], further indicating the severe undercount of suicides occurring behind prison walls.

Furthermore, the degree to which modifications in conditions of confinement contribute to suicides in prison remains unclear. For instance, even when using historical data on suicides, we cannot analyze the association between solitary confinement and suicides across systems, as most state policies and practices remain obscure with only three states providing data on whether an individual was housed in solitary confinement at time of death. It is well established that prison’s detrimental effect on mental health is elevated by time spent in solitary confinement [11, 22–25]. During the COVID-19 pandemic, the use of solitary confinement increased as it was a method utilized for transmission mitigation in the form of quarantine, medical isolation, and overflow housing [10, 26]. Thus, there is a need for reliable, real-time data on policies and practices around conditions of confinement.

There is also a need for more information on how these data are documented and how transparent the data collection process is. Incarcerated individuals must disclose their mental health concerns to staff. Yet, disclosure of mental health concerns to staff often leads to stays in solitary confinement and other inhumane conditions [27]. Additionally, stigma often prevents individuals from declaring mental health concerns in prison and individuals have little to no access to treatment, further discouraging individuals from disclosing concerns [28, 29]. Thus, it is important to understand who documents and determines suicide attempts and completions in prisons.

## Conclusions

Reliable, real-time data on cause of death, specifically for suicide and conditions of confinement in time of death, are urgently needed. It is only through more comprehensive data that we can better understand the harms of the carceral system and advocate for change in the following ways. First, it will allow us to better advocate for non-carceral mental health staff to be involved in prison intake and housing assignment functions. These professionals would provide crucial and frequently omitted data on individuals’ mental health at intake and the conditions in which they are placed. Second, it will allow us to quantify self-injurious or suicidal behavior within the context of solitary confinement practices and to ultimately end solitary confinement as punishment. Lastly, documenting the harms of the carceral system allows us to move towards evidence-based solutions to community safety outside the criminal legal system via decarceration.
